# Preparing Root Canals for Filling*Extract from a paper read before the Cleveland Dental Society, which is published in full in the *Dental Summary* for April.

**Published:** 1918-06

**Authors:** W. G. Ebersole


### PREPARING ROOT CANALS FOR FILLING*

BY W. G. EBERSOLE, D.D.S.

The first things to do is to thoroughly open up the pulp chamber, using the utmost caution to avoid destroying the normal walls of the chamber until after successfully locating the opening into each root canal, after which proceed to cut away enough of the tooth substance to give access to the canal in the straight line with its long axis.

This is done by the employment of both chemical and mechanical means. I always use the sodium-potassium alloy as my first chemical or therapeutic agent. This alloy is an alkali with powerful oxidizing and saponifying properties. It has such a great affinity for moisture that if the humidity is very high, combustion takes place before it can be carried from the glass tube to the canals. If it is brought into contact with water or moist tissue it burns with great violence and gives off intense heat. It is the quickest-acting and most reliable root-canal sterilizing agent of which I have any knowledge.

The alloy comes in very small tubes, and must be handled with the utmost care to avoid deterioration in the tube from faulty sealing after using. The alloy is carried direct from the tube to the mouth of each canal by the use of a small twist broach, and carefully worked into the canal. Care must be employed to have the pulp chamber dry before attempting to use the alloy. The chemical action which takes place in the canals soon produces quite a little moisture. Do not attempt to dry the canal until after you have made a number of applications of the alloy and then you safely may remove the excess moisture which the chemical activity has produced.

After carrying the agent into the canal for some dis-

* Extract from a paper read before the Cleveland Dental Society, which is published in full in the *Dental Summary* for April.

tance and waiting for a few moments, you may begin your exploration of the canal, using what I am pleased to call a Pathfinder, which is a very fine, smooth broach. This is used with a careful pushing and picking motion. If the broach is stopped, remove and curve the end a little and then insert and by a careful twisting motion you will, in most instances, be able to pass the obstruction and reach the apical foramen. Following this opening to the apex, wipe out any excess moisture with a few fibers of cotton on a broach and again apply the sodium-potassium alloy. Make sure that you carry it well down toward the apex, but not through into the apical space; then let the case rest a few moments while the agent either oxidizes or saponifies all organic matter within its reach, both in the canal and the tubuli. It is my belief that this agent is far more effective in both the root canal and the dental tubuli than any other agent that we can employ. After a wait of a few moments I wash out all the saponified matter. For this purpose sterilized water may be used.

Normal saline solution and Dakin solution have been recommended, but the objection to them in root-canal therapy is the film of salts that they deposit on the walls of the canals. In my practice I use pyrozone as my cleansing and washing agent instead of sterilized water, because of its greater cleansing properties. I continue the washing processes until signs of stain disappear from the cotton fibers or the paper points which are used as a vehicle to carry and remove the cleansing agent. I then dry the canal, using paper canal points to remove the moisture. The canal then is flooded with sulphuric acid C.P. (chemically pure), using an old pair of pliers to carry it to the canal and then using a fine, smooth broach to pump it well down to the apical end. The acid is permitted to remain for a few moments, that it may enter the dental tubules as far as possible. In fact, I carry new acid into canal several times within the next few moments; then it is neutralized by a sterilized solution of sodium bicarbonate, using enough of the solution, pumping it well into the canal and given plenty of time. I use the full strength acid because of its high affinity for moisture and because of the greater violence of its chemical activity when neutralizing it, for I am using acid in this instance not for the purpose of enlarging the canal, but for two other purposes. First, to neutralize any of the sodium and potassium that remains unsatisfied; second, for the mechanical action of the gas formed during the chemical process, in emptying the dental tubules of any saponified organic matter that has not been removed by previous treatment.

Remember that I am not using the strong acid for its dissolving or softening action on the inorganic tooth substance, but chiefly for the purpose of assisting in emptying the dental tubules of any remaining organic matter that they may better be prepared for the reception of my filling materials. Following the use of the sodium-bicarbonate I carefully remove all traces of it, using pyrozone for this purpose. The canal is then dried, using alcohol and paper canal-drying points.

